# Vaginal stimulation enhances ovulation of queen ovaries treated using a combination of eCG and hCG

**DOI:** 10.1002/vms3.552

**Published:** 2021-06-16

**Authors:** Naoaki Yoshimura, Masayasu Taniguchi, Tsukasa Terazono, Tetsushi Ono, Mitsuhiro Takagi, Yoko Sato, Maki Hirata, Fuminori Tanihara, Takeshige Otoi

**Affiliations:** ^1^ Laboratory of Animal Reproduction Faculty of Bioscience and Bioindustry Tokushima University Tokushima Japan; ^2^ The United Graduate School of Veterinary Science Yamaguchi University Yamaguchi Japan; ^3^ Department of Biological Science School of Biological Science Tokai University Hokkaido Japan

**Keywords:** chorionic gonadotropin, ovulation, queen, ultrasonography, vaginal stimulation

## Abstract

Follicular changes throughout the oestrous phase have been poorly documented in queens because of the location and the small size of ovaries. We investigated follicular development in queens treated with a combination of equine chorionic gonadotropin (eCG) and human chorionic gonadotropin (hCG) and evaluated the effects of vaginal stimulation by a tomcat on ovulation induction. A hormonal treatment was administered using a simple crossover design. Four queens were administered 150 IU of eCG (day 1) and 250 IU of hCG on day 5 and 6. Half of the queens were mated with a vasectomised tomcat for 3 days after hCG injection. Ultrasound imaging of the ovaries clamped at a subcutaneous site was performed once a day from day 1 to 7, and on day 13, and the serum concentrations of oestradiol and progesterone were examined on day 1, 5, 7 and 13. The mean number of follicles gradually increased with the eCG treatment and decreased after hCG injection. The ovulation rate of follicles was significantly higher in the vaginal stimulation group (70.0%) than in the control group (42.6%). During the hormonal treatments, the serum concentration of oestradiol and progesterone did not differ between the two groups. Ultrasound imaging of the ovaries clamped at a subcutaneous site showed that eCG and hCG treatment promoted the follicular growth and corpus luteum formation, respectively. The combination of hCG injection with vaginal stimulation by a vasectomised tomcat enhanced the ovulation rate of follicles.

## Graphical abstract

Ultrasound imaging of the ovaries clamped at a subcutaneous site showed that the combination of hCG injection with vaginal stimulation by a vasectomised male cat enhanced the ovulation rate of follicles.

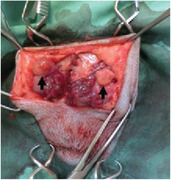

## 1 INTRODUCTION

Understanding the physiology of feline reproduction might be beneficial for breeding both domestic and wild felid species, as well as for biomedical research. The reproductive physiology of feline animals is particularly unique, compared to other animal species and is characterised by long‐day breeders. There is also spontaneous ovulation, but mechanical stimuli of the vagina by coitus induce ovulation under normal physiological conditions (Ferre‐Dolcet et al., 2020; Lawler et al., 1993; Shille et al., 1983). Thus, the vaginal stimulation by coitus is important for the occurrence of ovulation. In domestic cats, combination regimens of equine chorionic gonadotrophin (eCG) and human chorionic gonadotrophin (hCG) have often been used in artificial insemination (Roth et al., 1997; Swanson, Horohov, et al., 1995). eCG is typically administered first to stimulate follicular growth, and hCG is administered several days later to induce follicular maturation and ovulation. When combining an hCG treatment with vaginal stimulation, ovulation after natural breeding or artificial insemination is ensured (Roth et al., 1997). However, repeated treatment of eCG and hCG may cause an immunologically mediated refractoriness to ovarian stimulation (Swanson, Horohov, et al., 1995).

In queens, follicular changes (growth, ovulation, or atresia) throughout the oestrous phase have been poorly documented because successful ultrasound examination of the ovaries relies on the use of adequate equipment (high‐frequency probes) and experienced personnel. Thus, it restricts the progress made in our understanding of follicular development. It has been suggested that the current ability of ultrasound findings of ovaries is limited to follicular growth and ovulation during pro‐oestrus and oestrus (Malandain et al., 2011). Moreover, ultrasound echogenicity of corpora lutea is variable and depends on the observation period, thus making sonographic detection difficult (Gatel et al., 2020). However, when ovaries are clamped at a subcutaneous site, their follicular growth can more easily be monitored using ultrasound imaging (Hirata et al., 2018). Moreover, the ovarian clamp at the subcutaneous site can provide easy access for repeated collection of oocytes for in vitro fertilization in endangered animals.

The objectives of the present study were to evaluate the effects of vaginal stimulation by a tomcat on the induction of ovulation in queens treated with a combination of eCG and hCG, in which the ovaries were clamped at a subcutaneous site for ultrasound imaging of follicular growth.

## 2 MATERIALS AND METHODS

### 2.1 Animals

Four healthy domestic queens (aged 5–7 years; mean weight, 3.5 ± 0.5 kg) obtained from Kitayamarabesu Co. Ltd (Nagano, Japan) were used. The queens were individually housed in a single cage and were given standard commercial cat food once a day, and water *ad libitum*. The rooms where cages were placed were environmentally controlled with 12 h:12 h light:dark cycles at a temperature of 25–28°C. Laboratory animal management and experiments including anaesthesia protocol performed for surgery were performed with the approval of the committee based on the guidelines of the Laboratory Animal Committee of the Faculty of Agriculture, Yamaguchi University. The four cats used in the experiment had been treated for research purpose only and continue to be bred after the experiment.

### 2.2 Ovary clamp at the subcutaneous site

**FIGURE 1:**
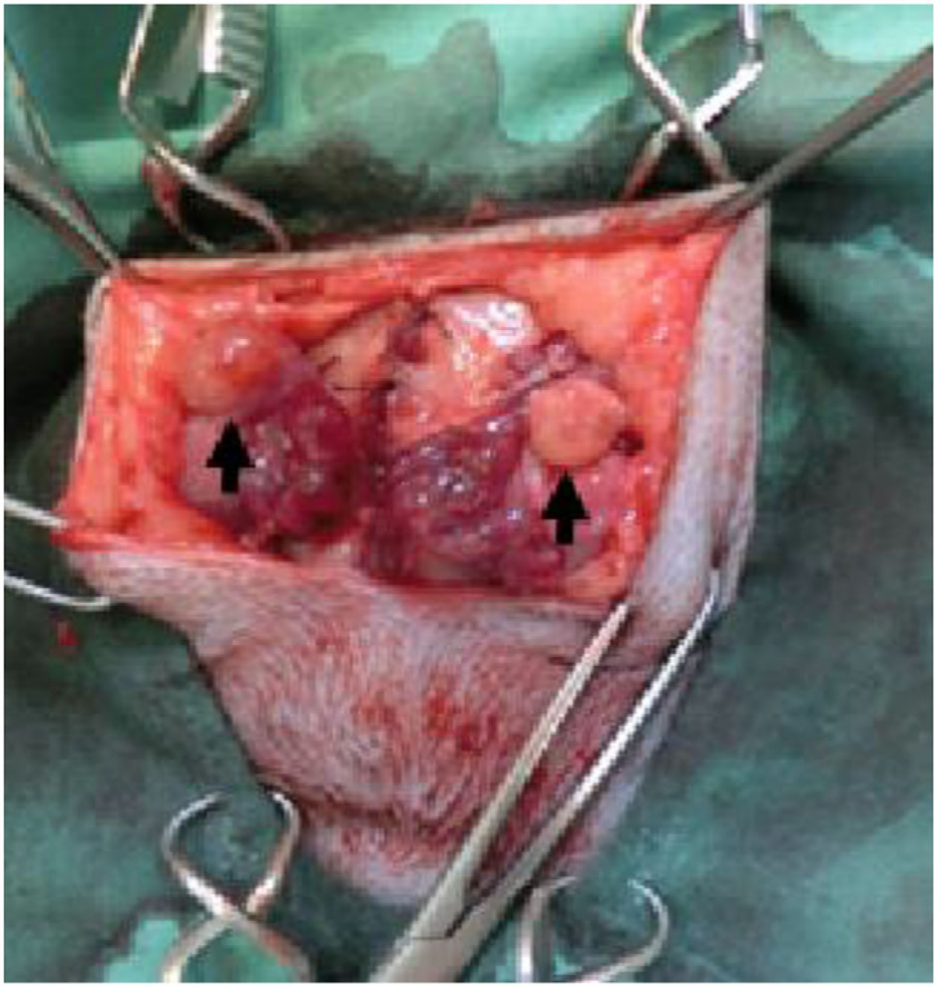
Gross morphology of ovary (arrow) placed on the external abdominal oblique muscle, making sure to not strangulate the ovarian blood supply

Bilateral malacotomy of four queens was performed using a ventral‐flank abdominal approach using routine techniques and materials, according to the methods described by Hirata et al. (2018). Briefly, the queen was mechanically ventilated with isoflurane in pure oxygen. Then, the uterine artery and vein were ligated and severed at the cranial tip of the uterine horn, after which the ovary was separated from the uterus. Each separated ovary, maintaining blood circulation from the suspensory ligament, was clamped at a subcutaneous site through the external abdominal oblique muscle. Each ovary was superficially placed on the external abdominal oblique muscle, making sure to not strangulate the ovarian blood supply (Figure 1). Finally, the subcutaneous layer and skin incision were closed.

### 2.3 Hormonal treatment and vaginal stimulation

To induce oestrus and ovulation, a hormonal treatment was conducted using a simple crossover design. Four queens alternately received the two treatments with and without vaginal stimulation by a vasectomised tomcat after hCG treatment at 2‐month intervals to approximate the normal interoestrous interval. Before the hormonal treatment, for each queen, ultrasonography results confirmed that the ovaries had no corpus luteum (CL). The queens were intramuscularly administered 150 IU of eCG (Kyoritu Seiyaku, Tokyo, Japan) (day 1). Each queen was given 250 IU of hCG (Kyoritu Seiyaku) on day 5 and 6 after the eCG treatment, with reference to a standard regiment with eCG and hCG in combination (Swanson et al., 1996; Villaverde et al., 2009). In the stimulation group, queens were mated with a vasectomised tomcat for 3 days after hCG injection on day 5.

### 2.4 Ovarian ultrasonography

**FIGURE 2:**
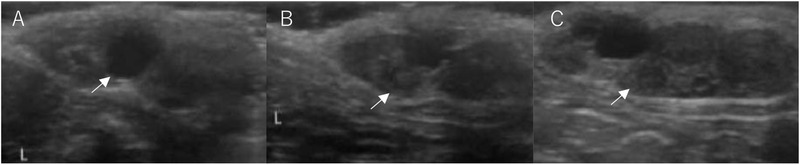
Ultrasonic appearance of the ovary clamped at the subcutaneous site on day 5 (a), 7 (b) and 13 (c) in the vaginal stimulation group. (a) The largest follicle (arrow) had a diameter of 5.8 mm. (b) The ovulated follicle (arrow) was observed after hCG treatment with vaginal stimulation by a tomcat. (c) Corpus luteum with 5.5 mm in diameter (arrow) is visible as a hypoechoic structure

Ovarian ultrasonography examinations were performed under sedation with an intramuscular injection of 1 mg/kg of ketamine hydrochloride (Daiichi Sankyo Co., Ltd., Tokyo, Japan). The development of the follicle and CL after inducing oestrus and ovulation was measured using an HS‐2100V veterinary ultrasound imaging system equipped with a 5.0–10.0 MHz linear array transducer (Honda Electronics, Aichi, Japan). The ovaries were examined once a day from day 1 to 7, and on day 13, and a diagram of all follicles with diameters ≥ 3 mm and the CL was recorded for each ovary (Figure 2). The follicles were divided into three groups, according to their diameter: small follicle group with a diameter ≥ 3 mm and < 4 mm, middle follicle group, with a diameter ≥ 4 mm and < 5 mm and the large follicle group, with a diameter ≥ 5 mm. The ovulation rates were calculated by dividing the number of CL observed on day 13 by the total number of follicles observed on day 5.

### 2.5 Hormonal assay

Blood samples were collected from the femoral vein on day 1, 5, 7, and 13 after eCG treatment (day 1); the samples were collected in a 2.5‐ml syringe under sedation and transferred into 1.5‐ml microtubes. The microtubes were centrifuged at 1,500 × *g* for 30 min. The serum was separated from clot and stored at –30°C until the sample was assayed for hormonal concentrations. The serum concentrations of oestradiol and progesterone were measured using a chemiluminescent enzyme immunoassay using commercial kits (Immulite 2000; Siemens Healthcare K.K., Tokyo, Japan).

### 2.6 Statistical analyses

Differences between queens with and without vaginal stimulation by a tomcat regarding the ovulation rates and hormonal concentrations were evaluated using an independent Student's *t*‐test by StatView software (Abacus Concepts, Berkeley, CA, USA). Differences with a *p* value of ≤ 0.05 were considered statistically significant.

## 3 RESULTS

**FIGURE 3:**
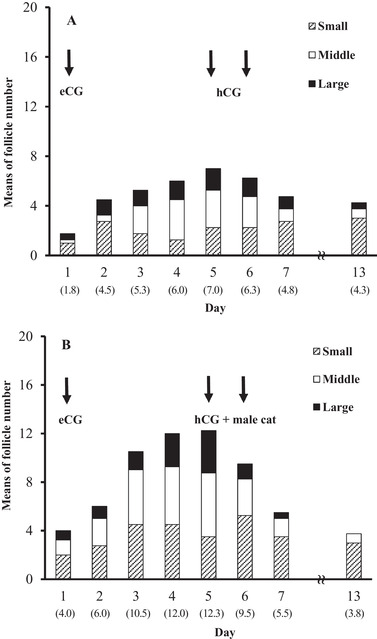
Profiles of total visible follicles in ovaries clamped at a subcutaneous site after eCG and hCG administration. Four queens alternately received the two treatments without (a) and with (b) vaginal stimulation by a vasectomised tomcat after hCG treatment at 2‐month intervals. Arrows indicate the time of eCG and hCG administration, and the onset of mating with a vasectomised tomcat. The ovaries were examined once a day from day 1 to 7, and on day 13 by an ultrasound imaging system equipped with a linear array transducer. The follicles were divided into three groups according to their diameter: small follicle (Small) group with a diameter ≥ 3 mm and < 4 mm, middle follicle (Middle) group with diameter ≥ 4 mm and < 5 mm, and large follicle (Large) group with a diameter ≥ 5 mm. The numbers within parentheses indicate the mean number of total visible follicles in ovaries

Follicles in ovaries clamped at a subcutaneous site were visualised as well‐defined, anechoic cavitary structures with diameters ≥ 3 mm. The mean numbers of follicles with a small, middle and large size gradually increased with the eCG treatment and decreased after hCG injection, irrespective of vaginal stimulation by a vasectomised tomcat (Figure 3). The total number of follicles on day 5 was significantly higher (*p* < 0.01) in the vaginal stimulation group (12.3 ± 1.0) than in the control group without stimulation (7.0 ± 0.7). In the vaginal stimulation group, moreover, a higher total number of CL was observed on day 13 (8.5 ± 0.5) compared with that in the control group (3.0 ± 0.4) (*p* < 0.01). The ovulation rate of follicles observed on day 5 was significantly higher (*p* < 0.01) in the vaginal stimulation group (70.0 ± 3.3%) than in the control group (42.6 ± 2.7%).

**FIGURE 4:**
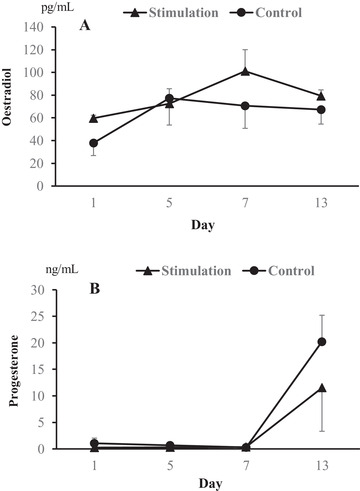
Serum concentrations of oestradiol (a) and progesterone (b) on day 1, 5, 7 and 13 after eCG treatment (day 1). Four queens alternately received the two treatments without (Control) and with vaginal stimulation (Stimulation) by a vasectomised tomcat after hCG treatment. Each bar represents the mean value ± SEM

As shown in Figure 4, the serum concentrations of oestradiol and progesterone during the hormonal treatments did not differ between the two groups. The mean concentrations of oestradiol in both the stimulation and control groups increased from 59.7 and 37.9 pg/ml (day 1) to 72.6 and 77.3 pg/ml (day 5) after eCG treatment, respectively. Moreover, the mean concentrations of progesterone also increased from 0.3 and 0.6 ng/ml (day 5) and 0.3 and 0.4 ng/ml (day 7) to 11.5 and 20.2 ng/ml (day 13) due to the hCG injection and both the hCG injection and vaginal stimulation, respectively.

## 4 DISCUSSION

Ultrasound is widely used in the screening of pregnant companion animals. However, a detailed ultrasound description of follicular growth in queens has been far less reported than that of other species, such as cows for instance (Adams et al., 2008). Transabdominal ultrasonographic examination of the reproductive tract in queens has been performed from the ventral abdomen in dorsal recumbency under sedation (Gatel et al., 2020). Recently, high frequency probes were used to study the ovaries, and the relatively small size of follicles could be measured (Gatel et al., 2020; Malandain et al., 2011); however, the ultrasound detection rate from the ventral abdomen has been reported to be good for follicles, but poor or moderate for CL, even when high‐frequency probes are used (Gatel et al., 2020). In the present study, therefore, the growth of follicles and CL was observed using ultrasound imaging of the ovaries clamped at a subcutaneous site. Furthermore, because ovulation could be induced by vaginal stimulation after the follicles reached a diameter of 3 mm (Malandain et al., 2011), the number of follicles with a diameter of 3 mm or more was recorded, according to the diameter size. In a previous study, mating was performed on day 3 of natural oestrus in queens (Concannon et al., 1980). However, in the present study, oestrus was induced by eCG administration, and queens were subsequently mated with a vasectomized tomcat after hCG injection on day 5. As a result, we observed that eCG treatment increased the number of follicles, regardless of the specific follicle size, hCG injection induced ovulation of developed follicles, and some follicles have regressed without ovulation, irrespective of vaginal stimulation. These results were supported in part by the experiment of Ferre‐Dolcet et al. (2020) who reported that GnRH‐treated queens between the second and fourth days of oestrus had about twice ovulation rates compared with the untreated queens. However, we found that the combination of hCG injection with vaginal stimulation by a vasectomised tomcat increased the ovulation rate of follicles, in which the vaginal stimulation increased the ovulation rates by about 27%. Goodrowe and Wildt (1987) demonstrated that the excessive follicle number by porcine FSH (FSH‐p) treatment cannot be reduced with any of the hCG or GnRH treatments, but combination of hCG injection with copulatory stimuli synergistically enhances the ovulatory response of queens treated with FSH‐p. Therefore, our findings indicate that, in queens with vaginal stimulation, follicular development was initiated by eCG, whereas ovulation was presumably enhanced by both vaginal stimulation‐induced endogenous LH surges and hCG injection‐induced exogenous LH surges. However, our present results are not consistent with the findings of Roth et al. (1997) who reported that when the numbers of CL were observed in ovaries obtained by ovariohysterectomy, the combination of hCG injection and natural mating did not increase the ovulation rates in queens treated with eCG. It has been reported that the serum concentrations of eCG after intramuscular injection are highly variable in domestic cats with even similar body weights (Swanson et al., 1996). Moreover, Yu et al. (2010) have suggested that the dose of eCG influences ovarian activity and embryo production in queens. In the present study, the number of middle and small follicles in the vaginal stimulation group was slightly higher than that in the unstimulated control group, indicating that the number of follicles before the start of the hormonal treatment might affect the ovulation rates. Therefore, the discrepancy concerning the vaginal stimulation effect remains to be explained, but it might, in part, be due to the influence of eCG used for ovarian stimulation and the status of ovarian follicles.

When administered prior to hCG, eCG stimulates follicle growth and potentiates the response of early antral follicles to the intrinsic folliculogenic and luteotrophic effects of hCG (Swanson et al., 1997). In the present study, we observed that, in all queens, serum concentrations of oestradiol and progesterone increased after eCG treatment and hCG injection, respectively. However, despite the higher total number of follicles and CL found in the vaginal stimulation group, the serum concentrations of oestradiol and progesterone during the hormonal treatments did not differ from those in the non‐stimulated group. In a previous study, it was suggested that there is a lack of correlation between the serum oestradiol‐17 concentration and the presence of distinct ovarian follicles, whereas serum progesterone has a strong positive correlation with the CL mass (Swanson, Roth, et al., 1995). In the present study, we evaluated the concentrations of oestradiol and progesterone during hormonal treatment in four queens using a crossover design. In cats, an extragonadal origin has been suggested to affect progesterone concentrations due to stress induced by restraint and handling even under anaesthesia (Chatdarong et al., 2006; Howard et al., 1992). However, considering a fact that only a limited number of samples were used for evaluation of the hormonal concentrations, there might be no significant differences in the progesterone concentrations because of the small number of samples and unknown factors due to a repeated use.

In conclusion, the observation of follicular development in the ovaries clamped at a subcutaneous site using ultrasonography indicates that the combination of hCG injection with vaginal stimulation by a vasectomised tomcat may increase the ovulation rate of follicles. However, further studies are needed to clarify the hormonal concentrations and to determine the vaginal stimulation effects because of a small number of examined animals.

## CONFLICT OF INTEREST

The authors declare that there is no conflict of interest.

## AUTHOR CONTRIBUTIONS

Naoaki Yoshimura, Masayasu Taniguchi and Tetsushi Ono conceived the study and wrote the manuscript. Naoaki Yoshimura and Masayasu Taniguchi performed the majority of experiments. Tsukasa Terazono performed the ovary clamp. Tetsushi Ono, Masayasu Taniguchi and Yoko Sato performed hormonal assay and revised the manuscript. Maki Hirata and Fuminori Tanihara reviewed the manuscript. All of the authors read and accepted the manuscript.

## ETHICS STATEMENT

The authors confirm that the ethical policies of the journal, as noted on the journal's author guidelines page, have been adhered to, and the appropriate ethical review committee approval has been received. All the animals involved in this study received humane care in compliance with the Guide for the Care and Use of Laboratory Animals prepared by the Institute of Laboratory Animal Resources, National Research Council. All procedures were approved by the Animal Research Committee of Yamaguchi University.

### PEER REVIEW

The peer review history for this article is available at https://publons.com/publon/10.1002/vms3.552.

## Data Availability

The data that support the findings of this study are available on request from the corresponding author. The data are not publicly available due to privacy or ethical restrictions.
